# Editorial: Host Genetics in Viral Pathogenesis and Control

**DOI:** 10.3389/fgene.2019.01038

**Published:** 2019-11-08

**Authors:** Ping An, Ju-Tao Guo, Cheryl A. Winkler

**Affiliations:** ^1^Basic Research Laboratory, Molecular Genetic Epidemiology Section, Basic Science Program, Frederick National Laboratory for Cancer Research, Frederick, MD, United States; ^2^Baruch S. Blumberg Institute, Hepatitis B Foundation, Doylestown, PA, United States

**Keywords:** Genetic epidemiology, GWAS, HBV, HIV-1, host susceptibility, innate immunity, variant, viral infection

## Host Genetic Susceptibilty/Resistance to Viral Infections

Viral diseases contribute to substantial morbidity and mortality and remain a major threat to global health. Although vaccines are notably successful in the prevention of many infections, effective vaccines are not available for most viruses including the pandemic human immunodeficiency virus (HIV) and hepatitis C virus (HCV). Direct antiviral agents can efficiently inhibit the replication of certain viruses but generally do not provide sterilizing cures, with the notable exception of HCV infection. Emerging and re-emerging viruses have caused an increasing number of disease outbreaks in humans and animals. There is unmet medical need to further our understanding of viral pathogenesis to enable more effective control and prevention of viral infections. Elucidating virus–host interaction is an essential step toward this goal.

Viruses generally show variation in both acquisition susceptibility and clinical presentations, suggesting that viral pathogenesis is due to complex interactions between host and viruses. This variability following viral infection has been attributed to a variety of factors, including viral strain and sequence variation as well as age, immune status and genetic background of host. This Research Topic included 21 review or original articles focused on multiple aspects of virus–host interactions, utilizing myriad cellular, genomic, and omics-based approaches to identify key genes and pathways in host defensive and virus offensive strategies.

## Host Proviral Factors

Viruses are obligate intracellular parasites that depend on host cellular functions for almost every step of their replication cycle. Host cellular factors required for viral replication, *i.e.*, host proviral factors (HPF), are usually identified through loss-of-function genetic screening approaches. By utilizing RNAi knockdown of host cellular gene expression in an Ebola virus infection cell model, Yu et al. validated 11 host proteins that support viral replication by testing for their interaction with viral proteins or RNA. Using a combination of pharmacological and genetic approaches, Yang et al. revealed that swine transmissible gastroenteritis virus induced diarrhea by interacting with its cellular receptor, epidermal growth factor receptor (EGFR), to weaken the Na+/water absorption ability of the Na+/H+ exchanger protein in small intestine epithelial cells. An EGFR inhibitor reduced viral proliferation and restored Na+ absorption. Li et al. identified novel host proviral factors required for the replication of enterovirus A71 that causes hand, foot and mouth disease by immunoprecipitation and mass spectrometry methods.

Due to the limited success of direct antiviral agents in curing viral infections and lack of antiviral therapeutics for emerging viral infections, considerable efforts have been expended to identify HPF vulnerabilities for antiviral drug development. Thus far, an antagonist of HIV-1 cellular coreceptor C‒C chemokine receptor type 5 (CCR5) has been approved by the US Food and Drug Administration for treating HIV-1 infection. Myrcludex B, a lipopeptide that specifically inhibits Hepatitis B virus (HBV) and hepatitis D virus (HDV) entry into hepatocytes *via* their cell surface receptor, is currently in clinical trials for treatment of HBV and HDV infection (Blank et al., 2016). In this Topic, Tan et al. reviewed the evidence that actin-binding protein non-muscle myosin II (NM II) is upregulated in diverse viral and bacterial infections and its involvement in multiple steps of viral replication, revealing the potential of NM II inhibitors for treating the microbial infection. Generally, targeting HPFs for essential steps of the viral lifecycle could provide broad spectrum inhibition against different families of viruses which share the same cellular factor, although overcoming potential toxicity may prove challenging (Chang et al., 2015). Unlike direct antiviral agents that are subject to the emergence of drug resistant viruses, particularly under the condition of monotherapies, host-targeting antivirals antagonizing HPFs are less likely to induce drug resistance due to the higher genetic barrier (Zeisel et al., 2013). Kumar et al. reported that a small molecule inhibitor of a cellular Ca2^+^ ATPase gene can inhibit the replication of two paramyxoviruses by blocking viral entry as well as biosynthesis, suggesting it as a novel target of host-targeting antivirals.

## Genetics of Innate Immune Control of Viral Infection

Innate antiviral immunity provides the first line of defense against invading pathogens by sensing pathogen-associated molecular patterns through pattern-recognition receptors. For instance, activation of cytoplasmic DNA sensor cyclic GMP-AMP synthase (cGAS) triggers interferon (IFN) production to defend against DNA viruses and retroviruses (Gao et al., 2013), which is also essential for IFN induction in mousepox infection (Chang et al.). IFNs restrict viral replication through induction of hundreds of IFN-stimulated genes (ISGs) that mediate antiviral effector functions (Chan and Gack, 2016). Genetic variation in the multiple innate immune pathway genes that elicit antiviral effector functions affect the fate of viral infection.

Among ISGs, interferon-induced transmembrane proteins (IFITMs), expressed at the plasma membrane and membrane of endocytic vesicles, restrict the infection of many different enveloped viruses by inhibiting the fusion of viral envelop with cellular membranes. Zhao et al. systematically reviewed the structural function relationship of IFITM proteins and natural genetic variations associated with acquisition and pathogenesis of viral infections. For instance, *IFITM3* variants that reduce gene expression or encode truncated protein are associated with higher risk to influenza infection and more severe clinical course. Host cells use 2′-5′-oligoadenylate synthetase (OAS)/Ribonuclease L (RNase L) to degrade viral RNA and/or induce IFN production *via* retinoic acid-inducible gene I (RIG-I) to defend RNA viruses. Ron et al. investigated how avian and mammalian OASL (OAS like) differentially inhibited the replication of a broad range of RNA viruses *via* these two pathways. Moreover, Rohaim et al. showed that transgenic chickens expressed IFN-induced protein IFIT5 have reduced pathology and virus shedding, providing proof of principle for developing genetically modified chickens with enhanced innate immunity for viral prevention.

MicroRNAs (miRNAs), regulating the expression of genes post-transcriptionally, are also effective in regulating the expression of immune response genes (Rodriguez et al., 2007). By integrative miRNA and proteome profiling, Khanduri et al. identified the top 10 miRNAs that regulate the major immune response pathways to the goat plaque-causing virus. Future integrative miRNA–mRNA–protein network analyses may identify key regulators of viral–host interactions.

Interestingly, An et al. showed a protective role of intranasal administration of IFN-λ to influenza A virus infection. By comparative transcriptomic and metagenomic profiling, Tan et al. demonstrated that an *in vitro* nasal system to influenza virus reflects the *in vivo* immune and metabolic microenvironment, thus suitable for translational development. Gendelman’s group compared the temporal and spatial host immune activation status in tissue compartments of HIV-1 infection in chimeric humanized mouse models transplanted with hematopoietic stem cells or mature human peripheral lymphocytes (Su et al.). Based on this line of work, Gendelman and collaborators reported that a combination of long-acting antiretroviral therapy (ART) and CRISPR-Cas9 for excision of integrated proviral DNA in the host genome successfully lead to permanent HIV-1 eradication in humanized mice (Dash et al., 2019). This work has important implications for curing HIV-1 infection in humans.

## Identifying Viral Restriction Genes by Genetic and Omic Epidemiological Approaches

HBV and HIV, which affect millions of people worldwide, contribute to substantial morbidity and mortality, and have no cure. To identify host genes that modify viral infection, several genome-wide association studies (GWAS) have been performed identifying genes associated with viral acquisition, disease progression, and clinical outcomes. A recognized limitation of GWAS studies is the high propensity for false-positive associations, and many associations have not been replicated or validated in subsequent studies. In addition, small studies are underpowered to identify small effect size variants or those with low population frequencies. Several articles provided critical reviews of HBV, HIV and cytomegalovirus (CMV) human genetic association studies and summarized the evidence supporting implicated genetic variants; the consensus message is that omics-based approaches are needed to identify critical host genes and pathways involved in the infectious process and pathophysiological mechanisms. Zhang et al. summarized association studies of host genetic variation with HBV infection, clinical outcomes, therapeutic efficacy, and responses to vaccines. They provided an evidence-based categorization of SNP associations based on study power, replication, and functional validation, with the HLA-DP and DQ genes showing replication among different studies. A review by An et al. concluded that individual variance in development of HBV-related hepatocellular carcinoma (HCC) is multifactorial and attributable to HBV genotype and mutations, host predisposing germline genetic variations, the acquisition of tumor-specific somatic mutations, as well as environmental factors. Before precision medicine can be fully utilized in early diagnosis and prognosis of HCC, a deeper understanding of the interplay of viral, environmental, and host factors is required. A major knowledge gap identified by An et al. is the paucity of established germline variants and somatic mutations that drive tumorigenesis and their pathophysiology. De Re et al. studied multiple clinical outcomes in Italian patients infected with HCV and found that variants in *IFNL3* and *TLR2* are risk factors for HCV-related HCC; by comparison, in Asian patients the combination of *IFNL3* and *PD-1.6* markers better define the HCV-related outcomes, likely due to divergent variant distributions in the two populations and highlighting the need for genetic studies in diverse populations.

Despite multiple GWAS and meta-analyses, only *HLA* class I and *CCR5* variant alleles have been securely identified with HIV acquisition or progression to AIDS, suggesting that many more rare variants, with the potential for large effect sizes, or common variants with small effect sizes remain undiscovered. Tough and McLaren assessed the interaction of the host and viral genome and their influence on HIV disease. They estimated that 30% of variance is attributable to common heritable effects of host genetic variation. Viral sequence variability, shaped by both random mutations and the selective pressure of the human immune response (i.e. HLA protective epitopes), also influences disease progression, emphasizing the need to study HIV infection in the context of both host and viral genetic variation. Le Clerc et al. provide an overview of the results of large-scale “omics” technologies to identify host genes that contribute to HIV pathogenesis, including genotype association and functional genomic, transcriptomic, proteomic and epigenomic screens. The authors consider that the lack of signals by GWAS, beyond *HLA* and *CCR5*, are partially attributable to false negatives due to the statistical constraints (stringent multiple testing corrections) and the overall small sample size of most HIV GWAS studies. Moving forward, integrative analysis of big multi-omics datasets in a collaborative setting is key to capture the multidimensional complexity of HIV-1 pathogenesis and to reveal actionable targets for drug development.

CD8 T cells and natural killer (NK) cells are key players in the host immune response to viral infection, but the functions of these cells can be repressed by cell surface inhibitory molecules particularly, the killer cell immunoglobulin-like receptor KIR3DL1. HLA-Bw4 homozygosity has been associated with control of HIV-1 replication and protection from AIDS (Flores-Villanueva et al., 2001) and is carried in the two major HIV protective alleles HLA-B*27 and HLA-B*57. By quantifying multiple immune activation and response markers in acute HIV-1 patients carrying Bw4 homozygousity, Zhang et al. demonstrated that KIR3DL1^-^ CD8 T/NK cells, conferring stronger T cell activation and response, contribute to the control of early HIV-1 replication.

An et al. studied African-specific alleles in *APOL1*, encoding the trypanolytic Apolipoprotein L1 protein. The two coding alleles restore the ability of APOL1 to lyse African trypanosomes causing human trypanosomiasis in carriers at the cost of increased risk of kidney disease in homozgyotes (Genovese et al., 2010). Although *in vitro* evidence suggests that APOL1 restricts HIV by multiple mechanisms (Taylor et al., 2014), An et al. found no evidence that *APOL1* renal risk variants affected HIV-1 susceptibility, viral load and disease progression to AIDS. Sezgin et al. reviewed human genes involved in human CMV infection and related diseases including HIV-1 opportunistic infection. They highlighted the relationship of immunoglobulin (Ig) allotype variation and CMV antibody response and immune-modulating genes that effect susceptibility to CMV diseases.

## Conclusion

The articles in this Topic provide a comprehensive overview of the state of genetic and omic-based tools to elucidate the genetic architecture underpinning susceptibility to viral infections and the pathogenesis of viral diseases. Although omics-driven viral-host interaction studies are in their infancy, integrated omics-based investigations should reveal host factors that can be exploited for the prevention and effective treatment of viral infections.

## Author Contributions

All authors co-edited the Research Topic. All authors wrote, edited, and approved the final version of the Editorial.

## Funding

The project was supported by grants from the U.S. National Institutes of Health (AI113267), the Commonwealth of Pennsylvania through the Hepatitis B Foundation. This project has been funded in part with Federal funds from the Frederick National Laboratory for Cancer Research, National Institutes of Health, under contract HHSN261200800001E and by the Intramural Research Program of the NIH, National Cancer Institute, Center for Cancer Research.

## Disclaimer

The content of this publication does not necessarily reflect the views or policies of the Department of Health and Human Services, nor does mention of trade names, commercial products, or organizations imply endorsement by the U.S. Government.

## Conflict of Interest

The authors declare that the research was conducted in the absence of any commercial or financial relationships that could be construed as a potential conflict of interest.
